# A Mixed-up Letter

**Published:** 1858-10

**Authors:** 


### A MIXED-UP LETTER.

ONE of our subscribers tells us a great many things in a very few words; therefore, we like him. He has his own opinions, too; and seems to think that the most feasible plan for rendering the whole United States a free, undivided, prosperous, healthy, happy, and othodox people, is, for everybody else to "go the omnibus," and adopt his views. In the first place, he is of the "old school," *in toto.* To compress him into as few words as possible, he is an Allopathic, Calvinistic, Whig, slave-loving cotton-planter—a perfectly hopeless case! at an immeasurable distance from the kingdom—Mrs. BEECHER STOWE, PARKER, GARRISON, and others of like mind being judges. As proof that we do not exaggerate, we will give his own words: "I am a planter, owning seventy-five slaves." "I am an Old-School Presbyterian." "I hate demagogues, fanatics, and fools." From this last, we naturally infer that he must be a true blue Whig; as, according to our dictionary, "demagogue" and "democrat" are synonymous. Then he projects a tremendous medical question at us: "Doctor! Why don't you pounce on our medical schools for sending out such graduates as they are now doing, all over the country? It is a shame! I find the Philadelphia Colleges as much in fault as the others. Raise the standard of medicine—require a collegiate education, and a good character, and I will guarantee their success when they come among us." A pretty good suggestion, as coming from a layman; but there is a serious difficulty in the way. If medical diplomas were awarded only to young men of education, morality, and a high sense of honor, one college would hold all the "candidates" in the Union—our sixpenny medical schools would be turned into market-houses and railroad stations; and a young army of medical professors would have to break rock for the turnpike—with chance exceptions only: those "matriculate" who have the three essential requisites—one for "now," two for "then," to wit: the money to pay for the tickets, and the two qualities to insure success in getting a practice and keeping it—to say nothing and look wise. To cap the climax, and introduce the millennium in no time, he invites the North to come and see them, and make themselves better acquainted and more familiar with the practical workings of slavery: "then, what a glorious and harmonious nation we would have!" We know "both sides" and ends of slavery from personal observation and a close view, and can propose a better plan than colonization, abolition, or ELIHU BURRITT'S redemption. It is the hypothenuse route, the short cut, the expeditious direct path to peace, harmony, and prosperity. Let the multitudinous girls of the North, with tidiness, intelligence, and handiness, emigrate to the South, and marry the sons of planters; and let our young men, who have nothing to depend upon but education, energy, and enterprise, take under their wing the heiresses and rich widows—that's all. The plantation argument is perfectly irresistible—maid or minister, priest or politician, all bow before it with a docility and submissiveness perfectly amazing to anybody who hasn't found out that the seven principles rule the world, to wit: the five loaves and two fishes.

